# Array of stacked leaky-wave antennas in groove gap waveguide technology

**DOI:** 10.1038/s41598-021-81640-7

**Published:** 2021-01-26

**Authors:** Nafsika Memeletzoglou, Eva Rajo-Iglesias

**Affiliations:** Department of Signal Theory and Communications, University Carlos III, Madrid, 28911 Spain

**Keywords:** Electrical and electronic engineering, Physics

## Abstract

The design of an array of stacked leaky-wave antennas in groove gap waveguide technology is presented in this work. The proposed array is formed by simply stacking a number of leaky-wave antennas one on top of the other and feeding all of them with uniform amplitude and phase. The inter-element distance is studied in order to avoid grating lobes and to maximize the directivity. A feeding network based on vertical coupling is designed, where the input port feeds the bottom element, and then the energy is equally coupled to the other elements. To obtain maximum directivity the phase is corrected at each element separately. The central frequency of the proposed design is 28 GHz. With this technique of stacking the elements a pencil beam is achieved, i.e. the radiated energy is focalized in the two main planes. The designed array with four elements achieves an enhancement of + 5 dB, reaching 24.5 dBi of directivity in comparison to 19.6 dBi of directivity of the single leaky-wave antenna made in this technology. A prototype is manufactured and measured and its results are presented and compared with the simulations.

## Introduction

Gap waveguide technology is relatively new, but since its appearance it has been gaining popularity^1^. Basically it is a two dimensional metasurface structure, that generates a stop band for parallel plate modes^2^. In other words, it is a waveguide where the bottom metallic plate is replaced by a high impedance structure that acts like a perfect magnetic conductor (PMC). When above this surface, at a distance smaller than $\lambda /4$ a metallic plate (PEC) is placed, then no electromagnetic waves can propagate between the two surfaces. The PMC is made with a textured surface^3^ with periodic elements. This technology is a good example of the effective use of metasurfaces to design antennas and components^4,5^.

The first form of a gap waveguide was the ridge gap waveguide^6–8^ where to guide the EM waves along the structure a metallic ridge was used. Later on, other implementations were introduced^2,9–11^, one of them being the groove gap waveguide^12^. In this type of gap waveguide, the two lateral walls of a conventional rectangular hollow waveguide are replaced by rows of square metallic pins. The field is confined in the groove and the type of modes that propagate depends on the cross-section as for a conventional rectangular waveguide. The propagation inside a groove gap waveguide was studied in^13^.

The main advantage of this technology when compared with conventional rectangular waveguide, is that it can be fabricated in two pieces, which simplifies the manufacturing process, reduces the cost and no physical contact is required between the upper and the bottom plates of the waveguide when assembled together. In addition, the field mainly propagates in the air and consequently it exhibits low losses^14^.

Many applications have been presented using this technology, specially in the millimeter-wave bands. In 2016 the first leaky-wave antenna in groove gap waveguide was proposed in^15^. To form the antenna, the conventional groove gap waveguide is modified so that it allows the leakage of energy from one of the lateral sides. One side of the groove consists on pins that create the necessary band-gap, and on the opposite side of the groove, pins that leak the energy to the free space are placed. Therefore a leaky-wave radiating element is created based on the initial idea presented in^16^ but with more parameters to have a better control of the leakage i.e., height and width of the pin, their periodicity and the width of the waveguide. The radiation pattern of this type of antennas is dispersive, meaning that the direction of maximum radiation changes with frequency, feature that is useful in beam scanning applications^17–21^. To overcome the dispersive nature of the radiation patterns, a non-dispersive version of this antenna was first proposed in^22^, where the antenna was combined with a metamaterial prism also made with squared pins. The latter has also been developed with holes^23^ instead of pins in^24,25^ or with the combination of pins and holes in glide symmetry^26^. Leaky-wave antennas have been also proposed using other versions of the gap waveguide technology^27–30^.

In the aforementioned leaky-wave antennas using groove gap waveguide technology^15,17,20,22,24–26^, medium directivity levels are obtained (below 18 dB in most cases). Furthermore, all these antennas focalize the radiation mainly in one plane (the H-plane) therefore creating fan-beam radiation patterns. The purpose of this work is to create an array of these leaky-wave antennas with the aim of reaching higher directivity levels than a single leaky-wave antenna by obtaining a narrow beam in the two main planes, i.e. a pencil beam.

To form the array, leaky-wave antennas are stacked one on top of the other. To avoid grating lobes and to achieve maximum directivity, the distance of the stacked elements must be studied. In order to couple the energy uniformly in the different elements of the array, a feeding network is proposed. Furthermore, the phase of the radiating elements is corrected separately, in order to achieve in-phase radiation and maximum directivity by controlling the phase constant of each stacked waveguide. The idea of stacking waveguides will not add excessive complexity to the design as the use of gap waveguide technology allows this stacking without requiring any electrical contact between the different layers, just by simply screwing them.

This paper is organized as follows. First the design of the array is presented, consisting of two parts: the leaky-wave antenna design, and the feeding and phase shifters design. Afterwards, the full-wave simulations are presented and compared to the experimental results of the fabricated prototype. Finally, the main conclusions of this work are discussed.

## Array design

The design of the proposed array is focused in two main parts. In the first part, the design of the leaky-wave antenna in groove gap waveguide technology is developed and *n* elements are stacked one on top of the other to create the array. The second part of the design focuses on the development of the feeding structure that is formed by vertical couplers and phase shifters. The purpose of this feed structure is to provide a uniform amplitude and phase to the *n* stacked antennas in order to achieve maximum directivity. The central frequency of the design is 28 GHz.

### Stacked leaky-wave antennas

**Figure 1:**
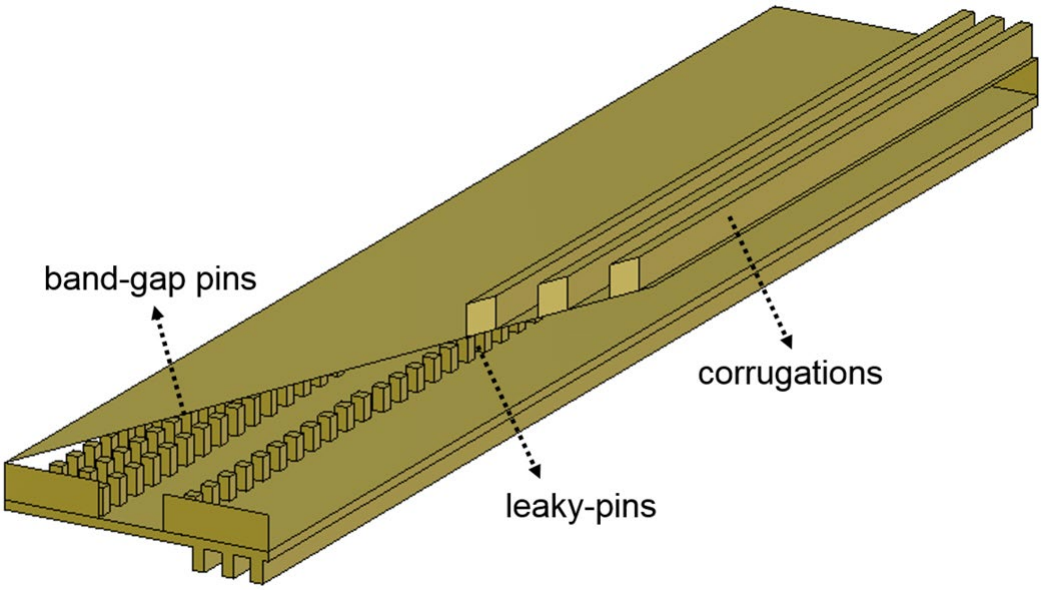
Model showing the leaky-wave antenna used as basic element for the proposed array. The model follows the example of the leaky-wave antenna proposed in^15^.

The *n* antennas of the array make use of the groove gap waveguide technology, following the design presented in^15^ as seen in Fig. 1. The groove is surrounded by square metallic pins. On one lateral side of the groove, the pins have specific dimensions so as to create the required electromagnetic band-gap (EBG pins). Three rows of EBG pins are enough to ensure that the field is confined in the groove, and no energy is leaking. On the other side of the groove, where the radiation is expected to ”leak”, the three rows of pins of the conventional groove gap waveguide are reduced to one row. The pins on this side have specific dimensions that allow the leakage of energy into free space. Above the metallic pins, at a distance smaller than $\lambda /4$ a metallic plate is placed. The dimensions (height, periodicity and width) of the pins on the radiating side in addition to the width of the groove, can control the leakage of energy from the groove gap waveguide to obtain a specific radiation pattern as made in^25,26^.

**Figure 2:**
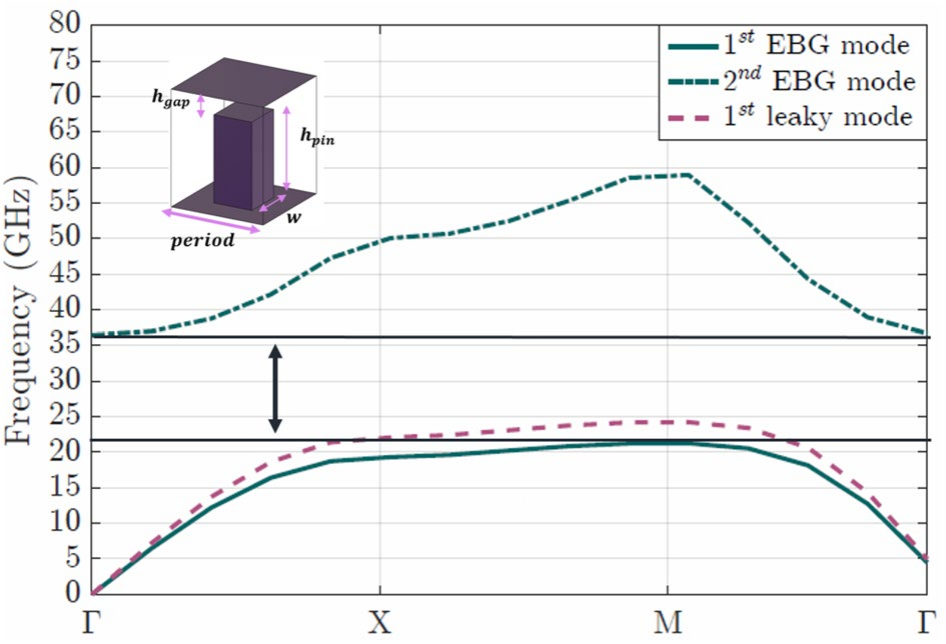
Dispersion diagram of the pin unit cell (shown in the inset) with dimensions $h_{pin}=$2.81 mm, $h_{gap}=$0.94 mm, $w=$1.31 mm and $period=$3.19 mm for the band-gap pins, while the leaky-pins have the same dimensions except for their height $h_{leaky}$=2.44 mm and the air gap $h_{gap,leaky}$=1.3 mm. Between the first and the second mode there is an electromagnetic band-gap from 21 GHz to 36 GHz.

To design the antenna for the aiming frequencies we first study the pin-unit cell (see inset of Fig. 2). The dispersion of the pins on the non radiating side was calculated, in order to ensure the required band-gap covering the targeting frequency of 28 GHz. After a parametric study of the band-gap as a function of the dimensions of the pins, we have chosen pins with height $h_{pin}$= 2.81 mm, width *w*= 1.31 mm, periodicity *period*= 3.19 mm and air gap $h_{gap}$= 0.94 mm. These dimensions and periodicity, create a band-gap from 21 GHz to 36 GHz, as shown in Fig. 2. These simulations were made with commercial software CST Microwave Studio.

The dispersion of the pins on the radiating side (leaky pins) has been also studied (Fig. 2). Their dimensions do not differ from the ones of the band-gap pins, with exception for their height that is $h_{leaky}$= 2.44 mm, and consequently the gap between the pin and the upper metallic plate changes to $h_{gap,leaky}$= 1.3 mm. As seen in Fig. 2 the lower limit of the band-gap for these pins is close to the operation frequency of the antenna. This, together with the use of just a row of pins will allow the desired leakage as demonstrated in^15^.

For a single antenna of this type, and using the same electrical length as in^15^, approximately 13$\lambda$, for a groove with similar dimensions to a standard WR34 waveguide but with a progressive change in the width (starting from 8.1 mm and ending with 7 mm) in order to control the attenuation constant making it smaller at the beginning of the antenna and progressively bigger at the end as in^15^, the directivity varies between 19.3 and 19.6 dBi from 27.4 to 28.6 GHz. If now two antennas are simply stacked and fed with the same amplitude and phase, the directivity can increase up to 22–22.2 dBi for the same frequencies. If a total of four antennas are stacked, the directivity reaches 25.8 dBi. These directivity levels, correspond to a spacing of 7.9 mm (0.7$\lambda _0$ at 28 GHz) between the antennas. This spacing is the result of a parametric study of the distance between the elements, that was changed by changing the thickness of the upper wall of the waveguides. More radiating elements of this type could be stacked to form an array with higher levels of directivity. For the purposes of this paper, a four element array was chosen to demonstrate the proposed methodology.

**Figure 3:**
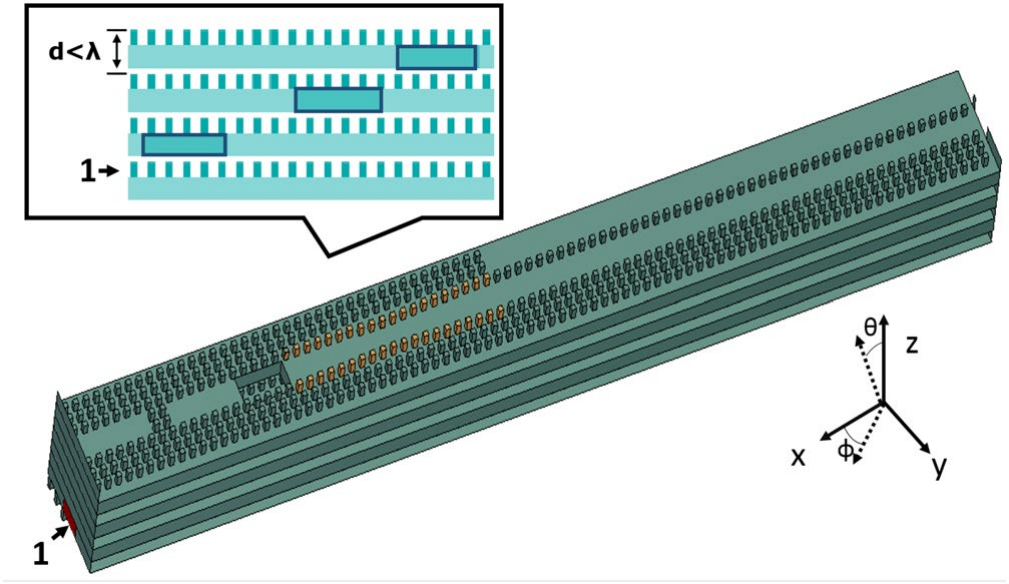
Perspective view of the array. In the inset a side view of the progressive manner the rectangular slots are placed. Number 1 indicates the waveguide feeding port. With orange are indicated the pins that are used to change the width of the groove, in order to correct the phase of each element before the radiating part of the array.

The inter-element distance is defined by the thickness of the upper broad wall of the waveguides, that separates the antennas, plus the height of the the waveguide itself (see the inset of Fig. 3). To avoid the appearance of grating lobes, the spacing between the elements should be less than $\lambda _0$ at the operating frequencies. Since the air gap and the pin height are fixed to achieve the desired band-gap following the example of the antenna designed in^15^, the inter-element distance is controlled only by the thickness of the broad walls. Later in this paper, the distance between the antennas is taken under consideration when designing the vertical coupler.

### Vertical coupler and phase correction

In order to study the number of elements to be stacked, the array was fed by *n* waveguide ports, directly connected to each element to obtain uniform feeding (same amplitude and phase). However, this is not a realistic scenario. In this direction we have designed a vertical feeding network that requires direct feeding by a standard WR34 waveguide transition only in one element of the array.

The aim of the proposed feeding network is to couple the energy uniformly to all the antennas to maximize the directivity. As indicated in Fig.3 the feeding is done at the first (bottom) antenna by a waveguide standard transition. The incoming energy is then vertically coupled through a network of rectangular apertures. These apertures are located at the broad wall that separates the radiating elements.

The coupling through these slots is a function of their parameters: width, length and distance from the edge of the waveguide. Furthermore, we have seen that the thickness of the broad wall, and consequently the thickness of the rectangular slots, strongly affects the coupling between the antennas. On one hand, for a smaller thickness, we achieve better coupling. On the other hand we have the constrain of the thickness that we have calculated in the previous section to have the desired inter-element spacing and the design of the coupler is adapted to this value.

**Table 1:** Dimensions of the coupling slots.

Coupling of antennas	Distance from the edge (mm)	Width (mm)	Length (mm)
1st and 2nd	5.1	8.3	15.3
2nd and 3rd	32.9	8.3	15.3
3rd and 4th	49	8.3	14.2

Dimensions of the slots used in the feeding network. The distance from the edge refers to the distance of each slot to the beginning of the groove gap waveguide where it is made.

**Table 2:** Phase shifters.

Antennas	$\delta \phi$	Width (mm)
1st	$137.17$°	5.8
2nd	0	8.3
3rd	$-56.56$°	6.8
4th	$31$°	6.4

Phase shift required for each antenna, and corresponding modified width of the groove. The phase of the 2nd antenna was taken as a reference to calculate the phase difference with the other antennas. All the phase shifters have the same length of 5.5 mm.

**Figure 4:**
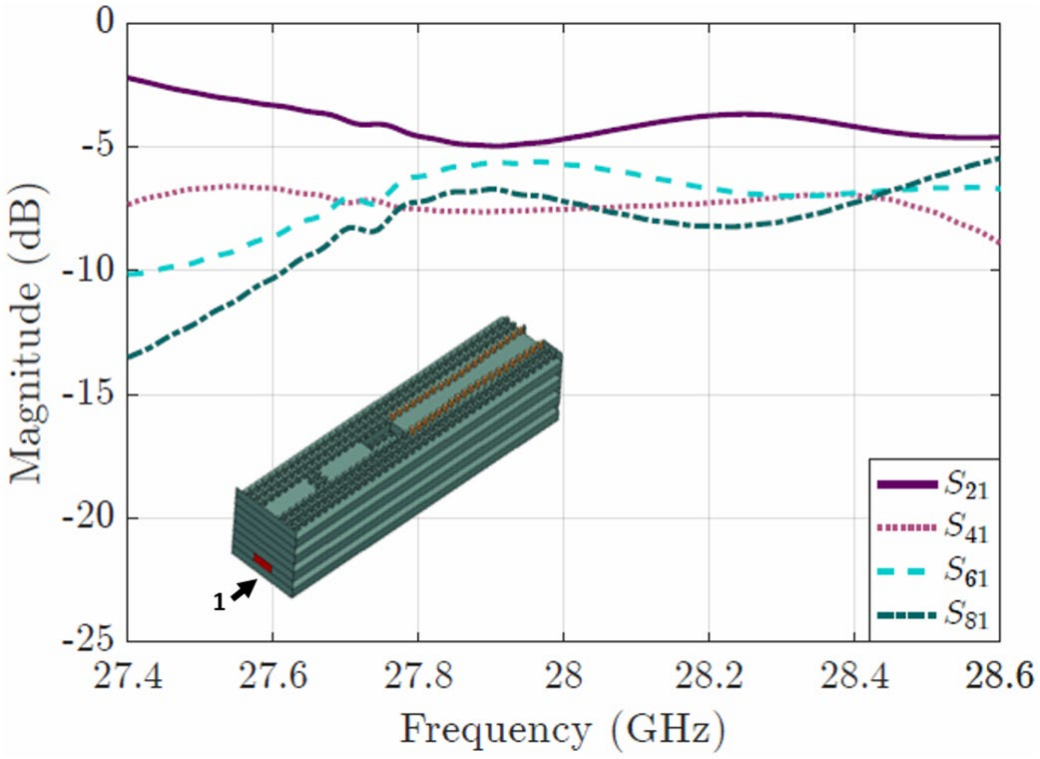
Coupling between the 4 elements of the array expressed in the form of S parameters. For the simulation, waveguide ports were placed at the end of the phase shifters at each of the 4 elements of the array. The $S_{21}$ parameter corresponds to the 1st (bottom) element, and respectively the $S_{41} , S_{61} , S_{81}$ correspond to the 2nd, 3rd and 4th elements. The inset shows the configuration of the feeding network and the phase shifters (orange pins).

**Figure 5:**
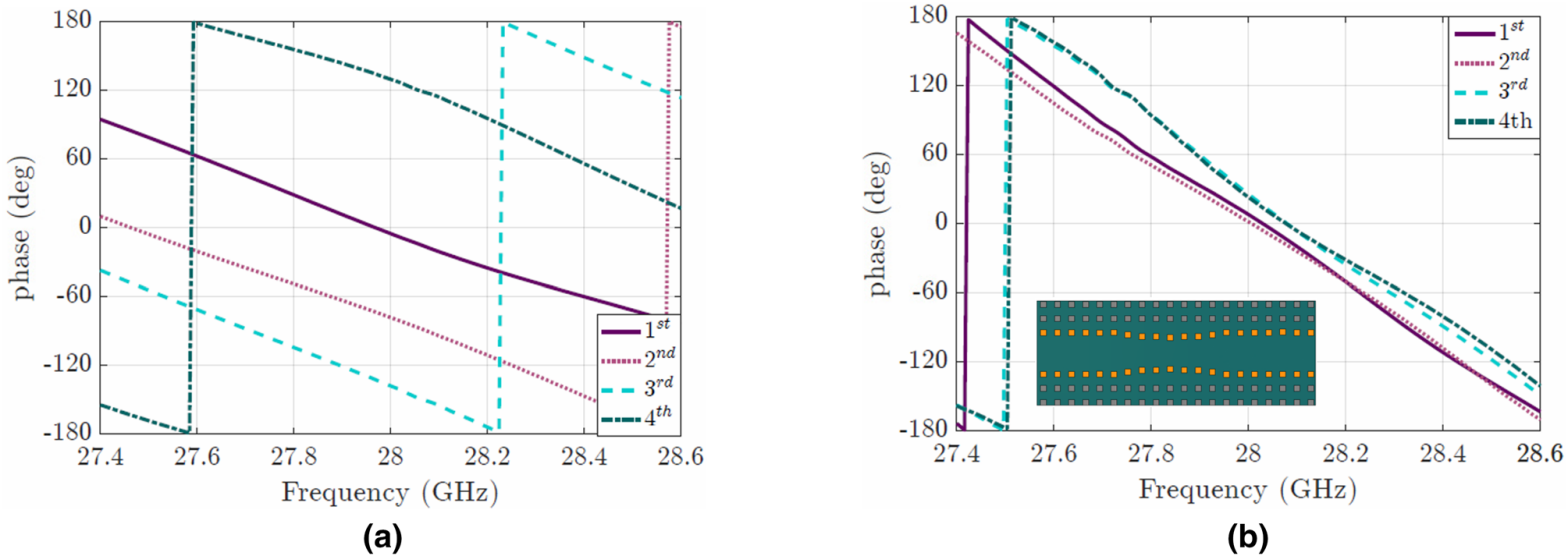
(**a**) Phase after the coupling network (**b**) Corrected phase after placing the phase shifters. In the inset, an example of a phase shifter made in groove gap waveguide technology is presented.

To study the coupling, standard rectangular hollow waveguides were stacked and a parametric procedure was followed in order to achieve uniform coupling. Three rectangular slots were used, with initial dimensions: length $\lambda _g$ at 28 GHz, width $\lambda _{g}/2$ and distance to the beginning of the waveguide $\lambda _{g}/ 4$, where $\lambda _g$ is the guided wavelength and is equal to 13.66 mm. As a first approach, the slots were aligned one on top of each other. Later we moved them to create a progressive coupling as seen in the inset of Fig. 3, and their initial dimensions were varied, resulting into the ones shown in Table 1. Once the vertical coupler was designed, the walls of the rectangular waveguides were replaced by pins to create the groove gap waveguide. The result of the coupling is presented in Fig.4, where approximately equal coupling is obtained within the band of interest.

Aiming to achieve maximum directivity, all the antennas should radiate in-phase. The incoming wave to the radiating part of the array after the designed coupler, should have the same phase in all the stacked elements. For this purpose, we designed phase shifters based on the pin arrangements. The width of each groove was progressively changed at each antenna by changing the position of the pins at both lateral sides of the waveguide. As a result the phase was corrected separately for each element, before the radiating part of the array.

**Figure 6:**
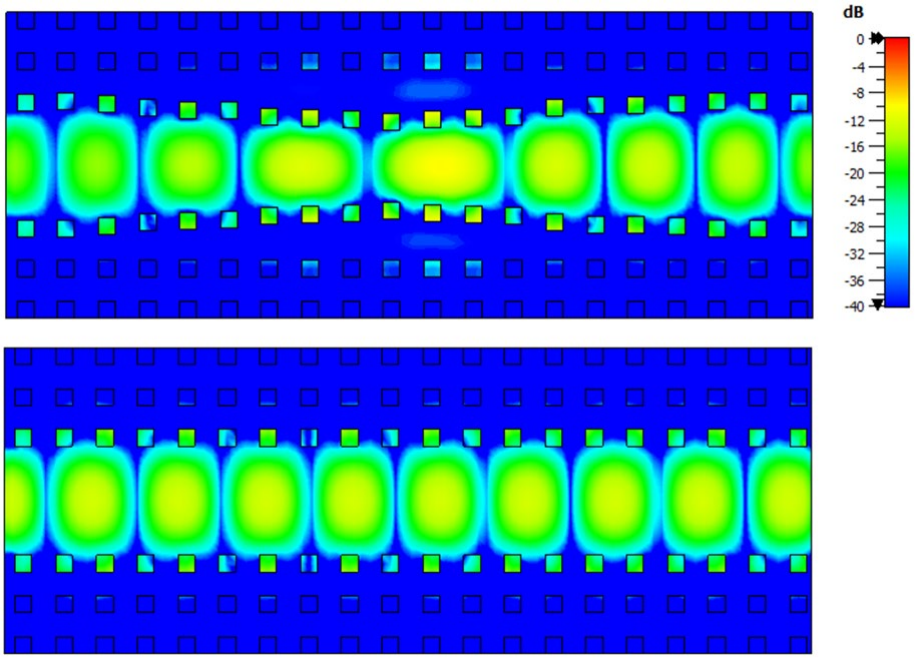
Electric field distribution for a phase shifting pin arrangement (up) and for a conventional gap waveguide made with pins (down).The color bar applies to both distributions.

First, we have calculated the phase difference between the elements for the output wave from the feeding network (see Fig. 5a). After calculating the required $\Delta \phi$, we have designed different phase shifters at each element as shown in Table 2. Apart from correcting the phase, these phase shifters must have good transmission coefficients close to 1. In Fig. 5b the corrected phases after the placement of the phase shifters are shown. In addition, an example of a phase shifter made in groove gap waveguide technology is presented in the inset of Fig. 5b. Finally in Fig. 6 an example of the phase correction using the modification of the pins is illustrated.

In the designed coupler and phase shifters, three rows of pins are used in the lateral walls of the groove gap waveguide to avoid any kind of leakage. These rows will be kept on the back (non radiating) walls of the leaky-wave antennas, whilst the radiating sides will have just one row as seen in Fig. 3.

The total length of each element of the array is 27.7 cm. The radiating part occupies 14.6 cm of the total length. The feeding network has a length of 7.4 cm, and the phase correction arrangement of the pins is 5.5 cm long.

## Results

In this section the results of the simulated antenna and the fabricated prototype are presented and discussed.

### Full-wave simulations

**Figure 7:**
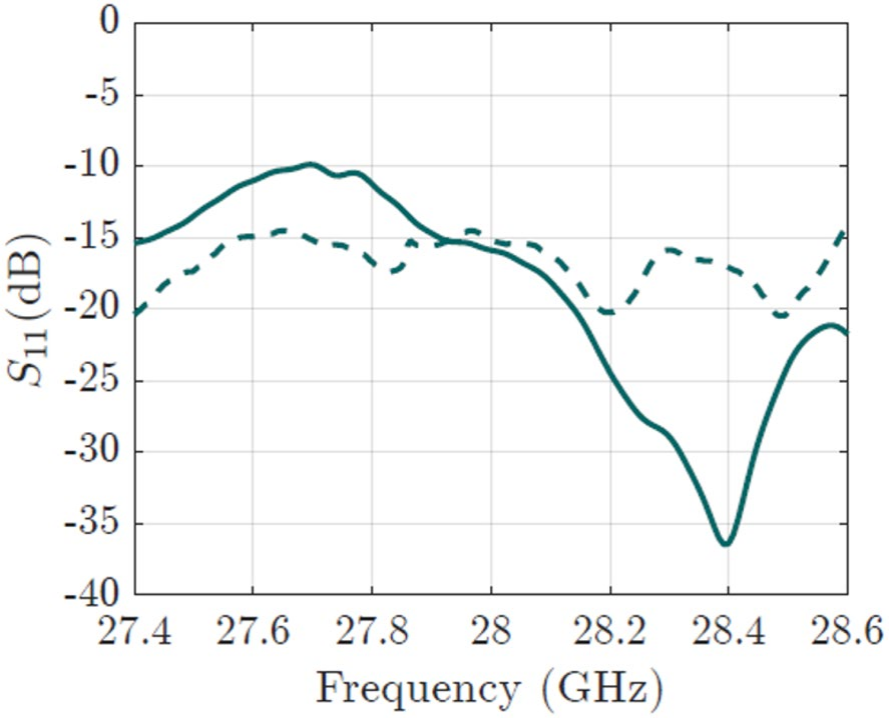
$S_{11}$ of the array as a function of the frequency. The solid line shows the simulated result and the dashed line the experimental one.

The proposed array was modeled and simulated using CST Microwave Studio. After designing the feeding and phase shifting network, we modeled the antennas and simulated the configuration shown in Fig. 3. The feeding is done only at the bottom antenna (denoted as “1”), and the $S_{11}$ parameter for this configuration is presented in Fig. 7. A good matching of the antenna is observed in the band of interest.

**Figure 8:**
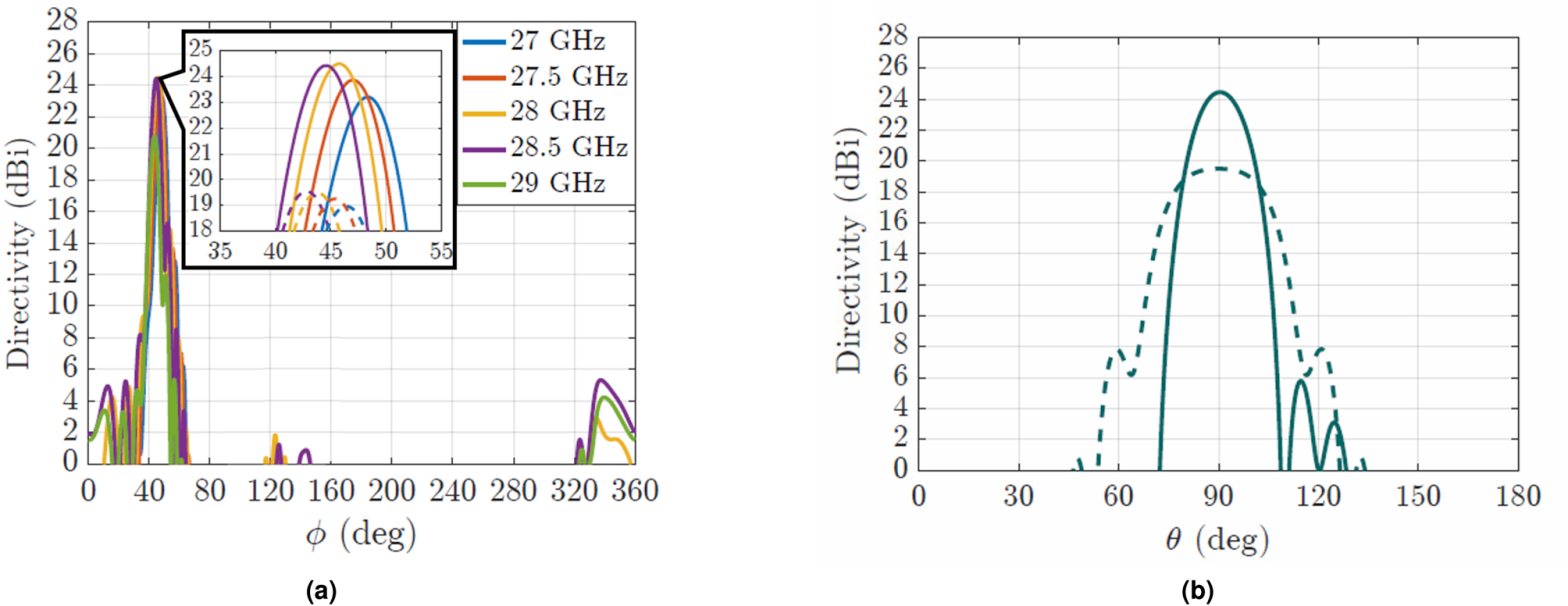
(**a**) Simulated directivity of the proposed array in the H-plane for different frequencies. In the inset, a comparison is made between the single antenna (dashed lines) and the designed array (solid lines). (**b**) Simulated directivity in the E-plane of the designed array at the central frequency of 28 GHz (solid line) compared to the single antenna also at the same frequency (dashed line).

Initially, a single leaky-wave antenna in groove gap waveguide was designed and simulated at 28 GHz. The obtained directivity values vary from 19 to 19.6 dBi as shown in the dashed lines of the inset of Fig. 8a. The enhancement of directivity provided by the designed four element array is shown in the same Fig. 8a with solid lines. The new value of directivity is 24.5 dBi at 28 GHz, reaching +5 dB more than the single leaky-wave antenna. The simulated realized gain of the array at the same frequency is 24.37 dBi.

**Figure 9:**
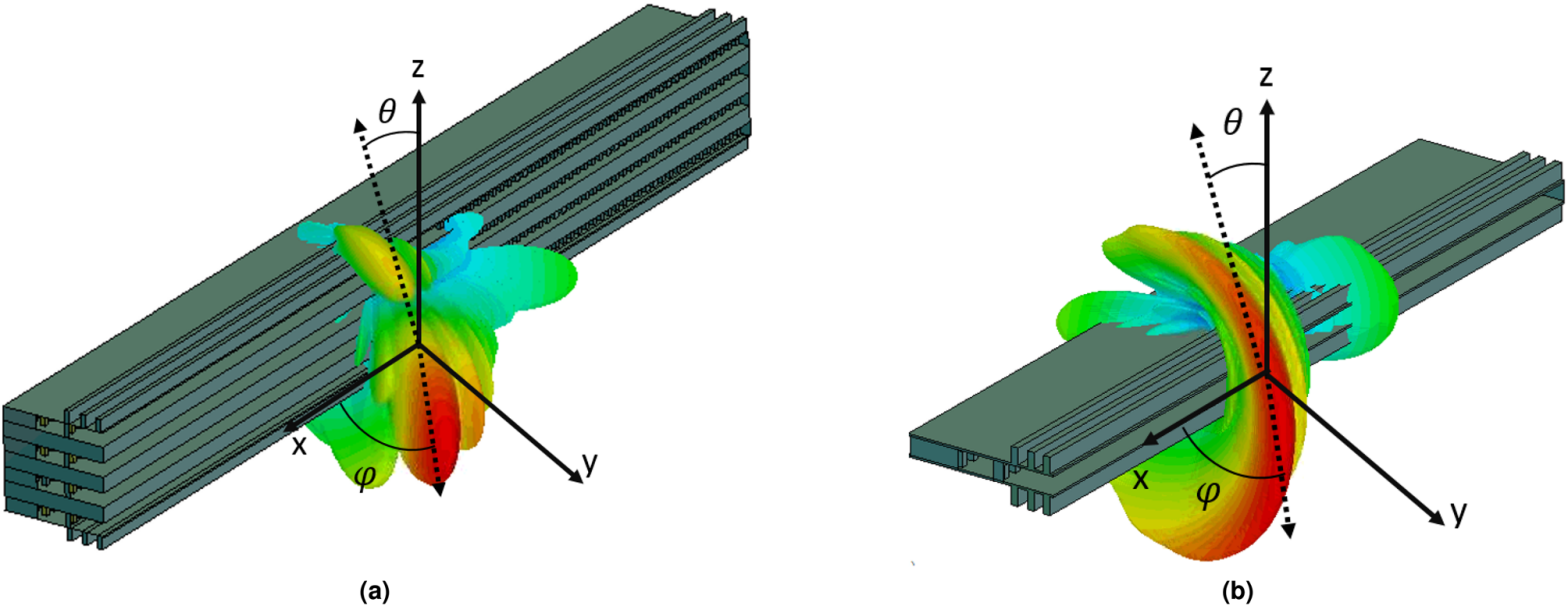
3D representation of the simulated radiation pattern at 28 GHz for (**a**) the proposed array and (**b**) the leaky-wave antenna that was used as basic element of the array.

Observing the radiation pattern in the E-plane of the array (Fig. 8b) at 28 GHz, it can be seen that the maximum is at broadside ( $\theta =90$) considering the plane defined by the direction of the main beam in the H-plane. Consequently, this proves that all the antennas radiate in-phase as desired. The result is also compared to the case of the single antenna (dashed line) in that plane. For both cases, array and single radiating element, the 3D radiation patterns are shown in Fig. 9. In this figure the transformation of the fan beam into a pencil beam is clearly seen.

**Figure 10:**
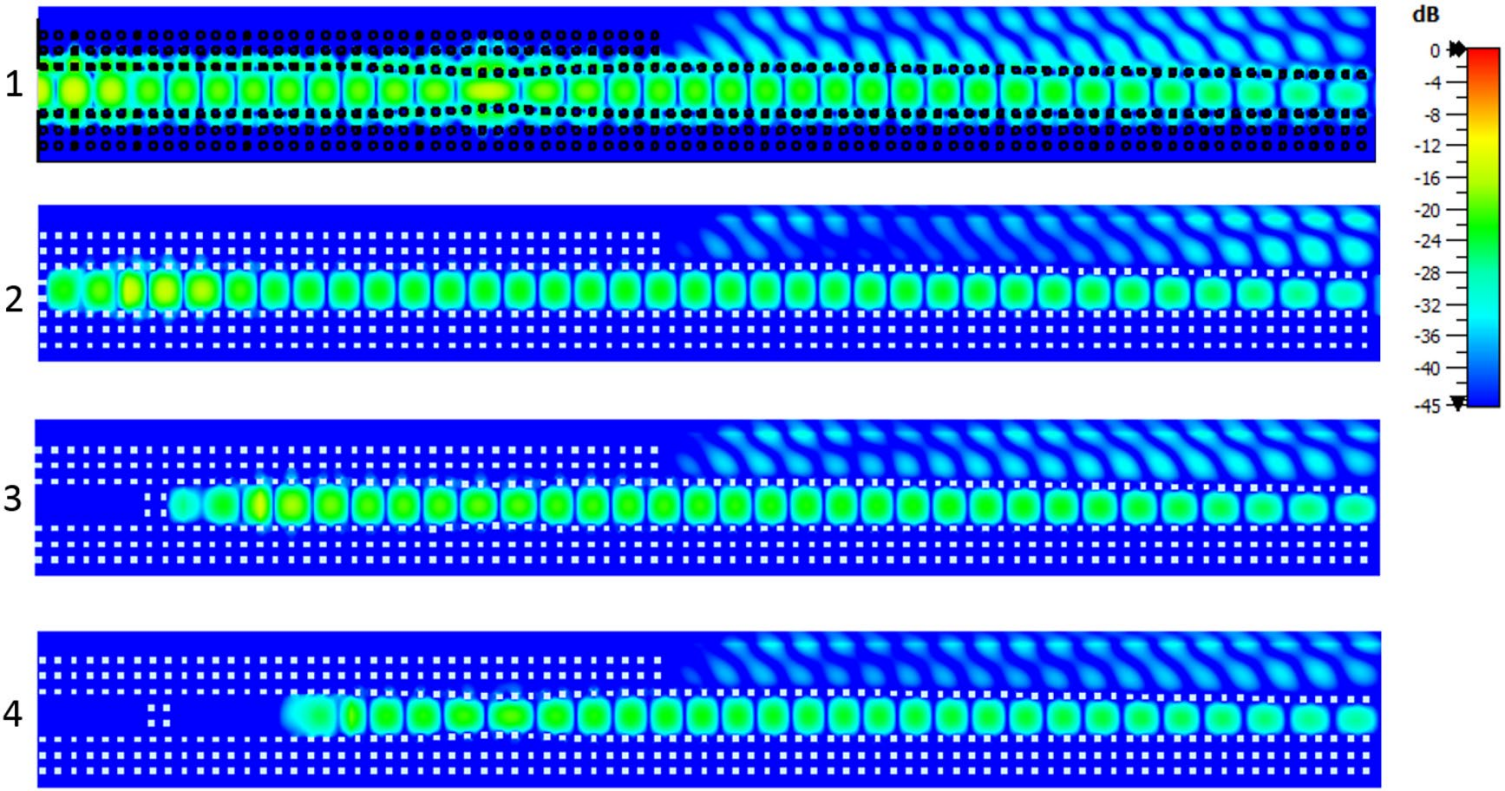
Amplitude distribution of the electric field of the proposed array shown separately for the four different elements. The bottom element is denoted as “1” and the top element of the stacked array is denoted as “4”.

The amplitude of the electric field at the central frequency 28 GHz is presented in Fig. 10 for the four radiating elements of the array. It can be seen that all the elements radiate with in phase and with approximately the same amplitude. In more detail in the same Fig. 10 it can be observed how the three phase shifters made with the pin arrangement affect the incoming wave at each level of the stacked array. At the second element (denoted as “2”), the pins do not form a phase shifter as this element was taken as reference for the phase difference. At the bottom element (denoted as “1”), where the required phase shift was significant (see Table 2) the manipulation of the wave is clearly evidenced.

### Prototype and experimental results

To validate the simulated results, a prototype was manufactured and measured in an anechoic chamber. The obtained results are presented in this section.

**Figure 11:**
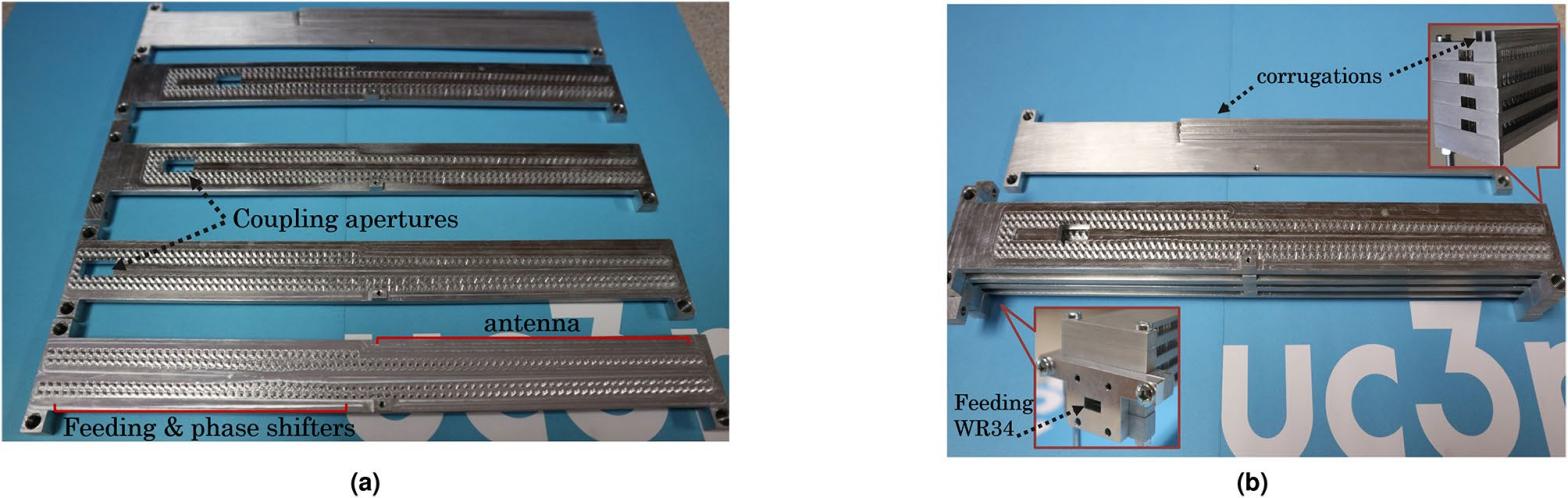
The fabricated prototype. In (**a**) the different elements of the array are shown separately. In (**b**) the antennas are stacked to form the array. In the insets, the front and back of the array is shown.

In Fig. 11a the four different elements of the manufactured antenna are shown, as well as the top metallic plate where corrugations are placed for the reduction of back radiation as usually made in this kind of antennas. To reduce the cost and the complexity of the fabrication, the band-gap rows of pins, were reduced to two. The effect of this reduction was verified in simulations and we have seen that even two rows are enough to ensure no leakage of energy. The end of each groove waveguide is terminated in open, to avoid the use of ports with matching loads at each element. To this aim, each groove leaky-wave antenna was slightly redesigned with a new changing in the width in order to modify the attenuation constant and further attenuate the wave that reaches the end of the antenna (as seen in Fig.10), so that no reflections are produced. Now the width changes from the initial 8.1 mm to 6.7 mm. This results in a transmission coefficient in the individual antenna ($S_{21}$) of about -44 dB that ensures that no reflections would be produced by the open end of the antenna.

In order to maintain the alignment of the stacked elements, a total of five screws were used at the front and back of the array, as well as in the middle. Thick borders at both ends of each element establish the required air gap between them, together with some pillars in the middle of the back non-radiating side of the antenna. Besides, a flange to connect the standard WR34 transition was added at the input port.

Fig. 11b shows a picture of the prototype with all the elements stacked one on top of the other and the insets show the front and back of the assembled array.

**Figure 12:**
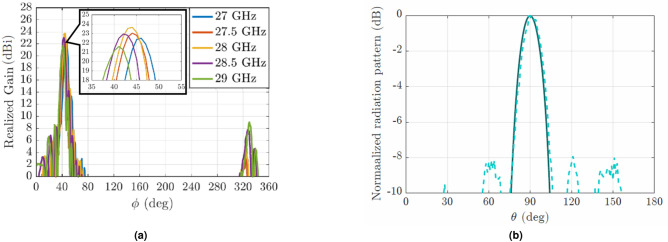
(**a**) Measured realized gain of the fabricated prototype for different frequencies. At the central frequency, the realized gain reaches 23.68 dBi of magnitude. (**b**) Normalized measured radiation pattern (dashed line) and simulated one (solid line) both corresponding to the E-plane at 28 GHz. The maximum is at $\theta$ = 90, proving that all the elements radiate in-phase.

In Fig. 7, the measured $S_{11}$ parameter is presented. Compared to the simulated return loss, the experimental result is maintained below −15 dB for all the frequency range of 27.4–28.6 GHz. The measured realized gain of the manufactured prototype is shown in Fig.12a. At the central frequency, 23.68 dBi of realized gain is achieved, being only 0.7 dB lower than the simulated gain. The overall variation of the gain goes from 21.6 to 23.68 dBi for the frequency range of interest.

Finally, in Fig. 12b the measured normalized radiation pattern in E-plane at 28 GHz is represented (dashed line) together with the simulated one (solid line) for comparison purposes. In both cases, the maximum is pointing at the broadside direction, confirming that all the elements radiate in-phase, and consequently proving the functionality of the phase shifters. The measured radiation pattern shows some side lobes that were not present in the simulation, however the overall agreement is very satisfying. The radiation efficiency of the array is high, reaching a value of 96.7% at the central frequency of 28 GHz.

## Conclusions

An array of four stacked leaky-wave antennas in groove gap waveguide technology has been presented. The proposed array achieves +5 dB enhancement in comparison not only to the single leaky-wave antenna presented previously in^15^, but also to other groove gap waveguide based leaky-wave antennas that are found in the literature^17,20,22,24–26^. It is the first time a pencil beam leaky-wave antenna designed in this technology is presented.

The formation of the array is done by stacking four leaky-wave antennas one on top of the other. The array consists of three parts: the coupling network followed by phase shifters that feed the radiating elements.

To feed the array, a network of rectangular slots was designed in order to couple vertically the energy approximately equal to all the antennas. The slots are located at the broad walls that separate the antennas and were placed in a progressive way with respect to one another.

The feeding network is then followed by phase shifters to ensure that the incoming wave to the leaky-wave antennas will be in-phase for all of the four antennas. The phase shifters were designed by changing the width of the groove. In gap waveguide technology, such a structure can be easily designed by changing the position of the pins that define the groove. For the whole length of the feeding network and the phase shifters, band-gap pins at both lateral sides of the waveguide ensure no leakage of radiation.

Right after the phase shifters, the radiation takes place in the leaky-wave antennas. As in^15^ the leaky-wave antennas have rows of band-gap pins on one side of the groove, that from three as in the original design, were reduced to two for manufacturing simplicity reasons, and on the other side one row of pins leaks the energy into the free space.

The experimental results validated the simulated array model. The measured realized gain is 23.68 dBi at 28 GHz. The array also focalizes the radiation in the E-plane, and the measured radiation pattern showed that the maximum is at the broadside direction, as seen in the simulated E-plane as well. This also proves that the phase shifters placed before the radiating part of each element, have the desired performance as seen in the simulated model.

This work is a proof of concept. The proposed methodology for the design of an array, could be implemented with other types of antennas in groove gap waveguide technology as the non-dispersive leaky-wave antenna presented in^22^. Also the number of stacked elements can be increased.

The advantages of the design are that it can be scaled to any frequency band since all the parts of the array are metallic providing good radiation efficiency, and it is all made using the same technology. The elements are simply stacked without requiring any kind of good metal contact.
